# Treatment of Alzheimer’s Disease and Blood–Brain Barrier Drug Delivery

**DOI:** 10.3390/ph13110394

**Published:** 2020-11-16

**Authors:** William M. Pardridge

**Affiliations:** Department of Medicine, University of California, Los Angeles, CA 90024, USA; wpardrid@ucla.edu

**Keywords:** blood–brain barrier, brain drug delivery, drug targeting, endothelium, Alzheimer’s disease, therapeutic antibodies, neurotrophins, TNF inhibitors

## Abstract

Despite the enormity of the societal and health burdens caused by Alzheimer’s disease (AD), there have been no FDA approvals for new therapeutics for AD since 2003. This profound lack of progress in treatment of AD is due to dual problems, both related to the blood–brain barrier (BBB). First, 98% of small molecule drugs do not cross the BBB, and ~100% of biologic drugs do not cross the BBB, so BBB drug delivery technology is needed in AD drug development. Second, the pharmaceutical industry has not developed BBB drug delivery technology, which would enable industry to invent new therapeutics for AD that actually penetrate into brain parenchyma from blood. In 2020, less than 1% of all AD drug development projects use a BBB drug delivery technology. The pathogenesis of AD involves chronic neuro-inflammation, the progressive deposition of insoluble amyloid-beta or tau aggregates, and neural degeneration. New drugs that both attack these multiple sites in AD, and that have been coupled with BBB drug delivery technology, can lead to new and effective treatments of this serious disorder.

## 1. Introduction

Alzheimer’s Disease (AD) afflicts over 50 million people world-wide, and this health burden costs over 1% of global GDP [1]. The dementia of AD correlates with the deposition of amyloid-beta (Abeta) in brain [2,3], which form fibrils and plaques in the extracellular space of brain. In addition, tau fibrils and aggregates form in brain leading to intracellular neurofibrillary tangles [4]. AD is believed to be a state of chronic neuro-inflammation [5], which may trigger the progressive formation of Abeta or tau fibrils and aggregates, which leads to dystrophic neurites. New treatments of AD are aimed at suppression of the Abeta and/or tau aggregates, suppression of the neuro-inflammation, and repair of the dystrophic neurites. The underlying pathology of AD has been known for many years. Despite the enormous global effort to develop new drugs for AD, there has not been an FDA approval of a new therapeutic for AD since 2003 [6].

The theme of this review is that the blood–brain barrier (BBB) is the singular reason for the lack of new treatments of AD. First, >98% of all small molecule drugs do not cross the BBB, and ~100% of biologic drugs do not cross the BBB [7]. Second, industry has been slow to develop BBB drug delivery technology that can be translated to human clinical trials. The profound under-development of BBB drug delivery technology is shown in a Pubmed analysis of the literature. As of October 2020, searching Pubmed for ‘Alzheimer’s disease drug development’ lists 9837 citations. Searching Pubmed for ‘Alzheimer’s disease drug development and blood–brain barrier drug delivery’ lists 191 citations, or 1.9%. Over half of these citations pertain to nanoparticles, which have proven difficult to translate to human neurotherapeutics, so that >99% of all AD drug development projects are conducted in the absence of BBB drug delivery technology.

One of the causes of the lack of development of BBB drug delivery technology within the pharmaceutical industry is the wealth of “BBB avoidance strategies.” These approaches to brain drug delivery are reviewed below, and lead the CNS drug developer into clinical trials of drugs that do not penetrate the brain, ending ultimately in a failed clinical trial. Another problem is that the measurement of BBB drug transport is not routine, and the methods that are used are prone to artifacts, as discussed below. Prior to a review of small molecule drugs in clinical trials for AD, the mechanism of small molecule transport across biological membranes such as the BBB is reviewed. Biologic drugs are large molecules that have minimal penetration of the BBB following systemic administration, as discussed below in Section 5. Yet, multiple biologics have entered AD clinical trials, and these are discussed. Finally, the re-engineering of biologics as BBB-penetrating IgG fusion proteins are reviewed in the context of a combination treatment plan for AD that attacks the disease at multiple levels, including neuro-inflammation, depletion of Abeta or Tau aggregates, and repair of dystrophic neurites.

## 2. Blood–Brain Barrier Avoidance Strategies

There are a number of BBB avoidance strategies that the CNS drug developer may adopt so as to enter a clinical trial without a BBB drug delivery technology [8]. As discussed below, these BBB avoidance strategies have not led to FDA approvals of new drugs for AD.

### 2.1. Drug Distribution in CSF Used as a Measure of BBB Drug Transport

**Figure 1:**
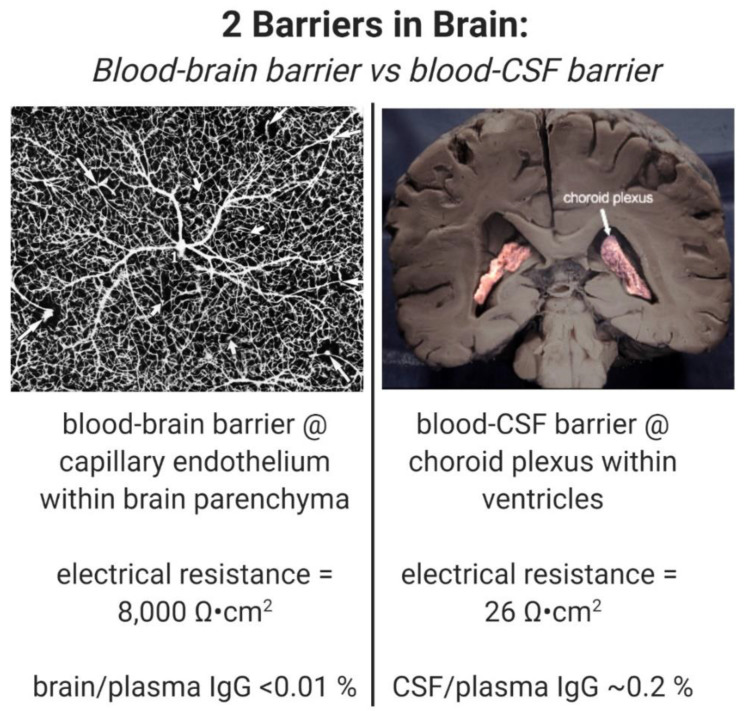
(**left**) Blood–brain barrier (BBB) is formed by the brain capillary endothelium which perfuse brain parenchyma, as shown in this inverted India ink study of the human cerebral cortex, which is adapted from [19] with permission. The BBB at the brain microvasculature is a very tight membrane barrier with an electrical resistance of ~8000 ohm·cm^2^ [11]. Owing to this tight barrier, IgG molecules do not cross the BBB, and the brain/plasma ratio of IgG is <0.01% [8]. (**right**) The blood–cerebrospinal fluid (CSF) barrier is formed at the choroid plexus lining the 4 cerebral ventricles, which include the 2 lateral ventricles shown here with pink coloration [20], and the third and fourth ventricles. Relative to the BBB, the choroid plexus is a leaky barrier with an electrical resistance of only 26 ohm·cm^2^ [10]. IgG molecules in plasma cross the leaky choroid plexus barrier and the CSF/plasma ratio of IgG is ~0.2% [12]. Image created with Biorender.com.

A drug is said to cross the BBB, because the drug is found to distribute into cerebrospinal fluid (CSF). However, drugs may enter into CSF, but not cross the BBB. This is because there are two barriers in the brain, as outlined in Figure 1. These are the brain capillary endothelial wall, which forms the BBB, and the choroid plexus, which forms the blood–CSF barrier [9]. The BBB, at the brain capillary, separates blood from brain interstitial fluid (ISF) within brain parenchyma, whereas the blood–CSF barrier, at the choroid plexus, separates blood from CSF. Drug transport into CSF is a function of drug delivery across the choroid plexus epithelial barrier, whereas drug transport into brain ISF is a function of drug delivery across the brain capillary endothelial barrier, which forms the BBB. The distribution of drug into CSF is used as a surrogate for measurement of drug penetration into the ISF of brain parenchyma, because it is assumed that CSF is in equilibrium with brain ISF behind the BBB. However, this is not the case, because the BBB and the choroid plexus are anatomically and functionally distinct barrier membranes [9]. The choroid plexus is >100-fold leaky compared to the BBB. As shown in Figure 1, the electrical resistance across the choroid plexus, 26 ohm·cm^2^ [10], is 300-fold lower than the electrical resistance across the BBB, 8000 ohm·cm^2^ [11]. Owing to the relative leakiness of the choroid plexus, all drugs in plasma distribute passively to the CSF in a manner inversely related to molecular weight (MW). For example, plasma proteins such as albumin and IgG freely move into CSF, and the CSF/plasma ratio for IgG is 0.22% [12], as shown in Figure 1. The finding of a ratio of IgG in *CSF*, relative to the plasma, of 0.2% is used to support the idea that the ratio of IgG in *brain*, relative to plasma, is also 0.2%, and therefore, there is a small, but significant transport of therapeutic antibodies across the BBB [13,14]. However, the distribution of a therapeutic antibody in brain tissue, not in CSF, should be used as a measure of antibody transport across the BBB. When brain tissue is measured, the brain/plasma ratio of a therapeutic antibody is ~0.01% [8], as shown in Figure 1. The use of therapeutic antibody distribution into CSF as a surrogate for BBB transport gives the false impression that the therapeutic antibody penetrates the brain, and validates the entry of the therapeutic antibody into AD clinical trials. As reviewed below, no therapeutic antibodies have been approved for the treatment of AD, despite the entry of >20 therapeutic antibodies into AD clinical trials.

In addition to the leakiness of solute transport across the choroid plexus, as compared to the brain capillary endothelium, these cellular barriers have different profiles of transporter expression [9]. P-glycoprotein, which is highly expressed at the capillary endothelium forming the BBB, is effectively not expressed at the choroid plexus forming the blood–CSF barrier [15,16]. Consequently, the co-administration of a p-glycoprotein substrate drug, and a p-glycoprotein inhibitor, produces increased uptake of the substrate drug into brain tissue, but has no effect on drug levels in CSF [17]. Expression of p-glycoprotein at the BBB in AD is unchanged compared to age-matched controls [18].

### 2.2. Role of Brain Blood Volume in Measurement of Brain Drug Uptake

A drug is said to cross the BBB, because there is a linear relationship between the dose of drug and the amount of drug detected in brain. However, this drug may be confined to the brain plasma volume, without any passage of the BBB. The confirmation of therapeutic antibody transport into brain is made by measurement of the brain/plasma ratio, which is a volume of distribution (VD) with the units of uL/gram brain. This VD should be compared to the brain blood volume, which is 10–20 uL/gram [21]. Therapeutic antibodies for AD have been administered in mouse models at low and high injection doses (ID), and the brain antibody concentration is higher at the higher ID [22,23]. However, this is the expected result if the antibody is confined to the brain blood volume, owing to lack of BBB transport, since the antibody concentration in blood is also higher at the high ID. The brain/plasma ratio of the antibody is 1–2 uL/gram [22,23], which is indicative of incomplete washout of the antibody from the brain blood volume, rather than actual transport of the therapeutic antibody across the BBB.

### 2.3. Drug-Induced BBB Disruption

The therapeutic anti-Abeta antibody, aducanumab, reduces brain amyloid plaque at higher injection doses (IDs), which is evidence for brain penetration of this therapeutic antibody [22]. However, these higher IDs of aducanumab also cause amyloid related imaging abnormalities of edema (ARIA-E) [22]. ARIA-E is a form of vasogenic edema that is associated with BBB disruption [24]. The published clinical trial data on aducanumab [22] shows there is a linear relationship between plaque reduction and ARIA-E [25]. A correlation of antibody-induced plaque reduction and antibody-induced ARIA-E suggests the antibody enters brain via a mechanism of BBB disruption. This antibody induced disruption of the BBB in AD parallels the BBB disruption and cerebral microhemorrhage in mouse models of AD following the administration of high doses of anti-amyloid antibodies [26]. Anti-Abeta antibodies that do not cause ARIA-E in AD also do not reduce brain amyloid plaque [25]. High doses of the anti-Abeta antibody, BAN2401, are said to cause less ARIA-E than aducanumab in patients with AD [27]. However, the assessment of ARIA-E with BAN2401 was made after only 4 months of treatment [28], whereas ARIA-E after aducanumab was assessed after 12 months of treatment [22]. If long-term treatment with BAN2401 and aducanumab are shown to produce comparable reductions in brain amyloid plaque, but BAN2401 causes less ARIA-E, then alternative mechanisms other than BBB disruption may account for BAN2401 entry into the brain. However, as discussed in Section 6.3 below, the penetration of the mouse homologue of the BAN2401 antibody through the BBB is minimal [29].

### 2.4. Transitory BBB Disruption

Enhanced BBB drug transport is possible in conjunction with parallel BBB disruption (BBBD) caused by the intra-arterial administration of hyperosmolar solutes [30], laser interstitial thermal therapy (LITT) [31], or the intravenous administration of micro-bubbles simultaneous with ultrasonic irradiation of the brain [32]. The latter approach to brain drug delivery in AD is presently in phase 1–2 clinical trials (NCT03119961, NCT03739905) [33]. The ultrasound/microbubble procedure causes reversible opening of the BBB to both small and large molecules by both increasing paracellular transport, via disruption of the endothelial tight junctions and increasing transcellular transport, via enhanced vesicular transport [32,34]. In addition to enhanced drug transport into brain, BBBD causes increased brain uptake of plasma proteins, such as albumin, which is toxic to neurons [35]. In preclinical models, BBB disruption by the intra-arterial administration of hyperosmolar agents causes chronic neurodegenerative changes in the brain [36], although it is not known if other forms of BBBD induce similar changes.

### 2.5. Small Molecule Biologic Mimetics

Neurotrophins were first proposed as treatments for AD over 30 years ago [37]. However, early neurotrophin clinical trials for neurodegeneration [38,39], which administered the drug by subcutaneous (SQ) injection, failed owing to the lack of transport of these large molecules across the BBB. An alternative strategy is the development of a small molecule neurotrophin mimetic [40]. The MW of such biologic small molecule mimetics invariably exceeds 500 Daltons (Da) in order to produce high target affinity. However, as discussed below, >98% of all small molecules do not cross the BBB owing to a MW threshold of ~400 Da. The development of BBB drug delivery technology for small molecules with a MW >400–500 Da is just as difficult as the development of biologics BBB delivery technology.

### 2.6. Brain Drug Delivery via Drug Injection into CSF

Drug injection into CSF is believed to reach the brain and bypass the BBB. For example, glial derived neurotrophic factor (GDNF) was developed as a neurotrophin treatment of a neurodegenerative disease, Parkinson’s disease (PD). So as to bypass the BBB, the GDNF was injected into the lateral ventricle via an Ommaya reservoir [41]. However, the clinical trial failed, and preclinical investigations showed the injection of neurotrophin into a lateral ventricle only distributes the drug to the ipsilateral ependymal surface of the ventricle, and not into brain tissue beyond ~1 mm from the CSF surface [42]. The limitations of drug delivery to brain via the CSF route was shown over 40 years ago, following the injection of small molecules into the lateral ventricle of the primate [43]. Drug distribution to brain from CSF is more uniform following direct injection into the cisterna magna, as compared to the lateral ventricle [44]. However, drug distribution into brain is limited to the CSF surface of the brain following the cisternal infusion of a 17 kDa single domain VHH antibody in rats [45]. The entire CSF volume is absorbed into the blood every 4 h in humans [9]. Owing to this rapid rate of CSF egress from brain to blood, there is also rapid movement of the drug from CSF to peripheral blood [9]. Drug injection into CSF is equivalent to a slow intravenous infusion of the drug [46].

### 2.7. Brain Drug Delivery via Drug Injection into Brain Tissue

The BBB was bypassed in PD patients treated with GDNF via direct infusion into brain tissue by convection enhanced diffusion. This trial failed with no beneficial effect in PD [47], and subsequent investigations in primates with this delivery system showed the brain concentration decreased logarithmically from the catheter needle tip, owing to drug movement into brain via diffusion, not convection [48]. In a failed clinical trial of neurotrophin gene therapy of AD, an intracerebral injection of an adeno-associated virus (AAV) encoding nerve growth factor (NGF) had no beneficial effect [49]. Subsequent post-mortem exam showed the diffusion of the NGF into the brain surrounding the injection site covered a distance <1 mm [50]. Therefore, the failure of GDNF in PD, or NGF gene therapy in AD, was not due to the lack of a therapeutic action of the neurotrophin, but rather was due to poor brain delivery of the biologic. Drug diffusion results in limited penetration of brain tissue, because diffusion decreases logarithmically with the diffusion distance [9].

### 2.8. Brain Drug Delivery via Trans-Nasal Drug Administration

Trans-nasal drug delivery is believed to reach the olfactory CSF and bypass the BBB. Actually, this route of administration is limited by a BBB-like membrane, the arachnoid membrane, which separates the nasal spaces from olfactory CSF [8]. Following intra-nasal instillation of the drug, the agent must first cross the nasal mucosal epithelial membrane. Once the drug enters the submucosal space, drug entry into the olfactory CSF is precluded by the high resistance arachnoid membrane. Therefore, the same principles apply to trans-nasal drug delivery as apply to BBB drug delivery. Lipid soluble small molecules with a MW < 400 Da cross nasal/arachnoid membranes via free diffusion. All other molecules only cross via either mediated transport, should the agent have an affinity for an endogenous transporter in the nose, or via nasal barrier disruption. The latter mechanism is the principal means by which drugs cross the nasal barrier following transnasal administration. The instillation of a volume >100 uL/nares in humans causes local injury and membrane disruption [51]. Positron emission tomographic (PET) imaging studies in rats show the drug is confined to the local nares following nasal administration [52,53]. A clinical trial of trans-nasal insulin in AD showed no benefit of this intervention [54].

## 3. Methodology of Blood–Brain Barrier Drug Transport

CNS drug developers need to know the extent to which the candidate drug crosses the BBB. The problem in the measurement of BBB drug transport is that there is no consensus methodology, and that several of the commonly used approaches have technical limitations.

### 3.1. In Vitro Models of BBB Transport

Brain capillary endothelial cells may be isolated and grown in cell culture to establish an in vitro model of the BBB. However, this 40-year old model has failed to replicate the transport properties of the BBB in vivo, owing to profound down-regulation of BBB tissue-specific gene expression in culture [55]. Even the best in vitro BBB models developed recently are still 100-fold leaky compared to the BBB in vivo [55]. Consequently, CNS drug developers cannot rely exclusively on drug transport across in vitro BBB models as a measure of drug entry into brain in vivo.

### 3.2. Cerebral Microdialysis

Owing to the limitations of using CSF as an index of BBB transport, direct sampling of brain ISF is made following the intra-cerebral implantation of a microdialysis fiber. The walls of such fibers are formed by porous dialysis membranes, which have a molecular weight cutoff (MWCO) of 20,000 Da, for microdialysis of small molecule drugs [56]. For cerebral microdialysis of therapeutic antibodies, a dialysis membrane with a 1,000,000 Da MWCO is used [57,58]. The problem with this approach is that the implantation of such probes into brain tissue causes local injury and BBB disruption. Albumin immunohistochemistry shows local BBB disruption around the fiber, and albumin entry into brain, for at least 24 hrs following the fiber implantation in brain [59], and astrogliosis around the implant is found at 3 days following fiber insertion in brain [60]. The brain uptake of small molecules such as sucrose, which has minimal transport across an intact BBB, is enhanced for at least 10 days following fiber implant [61]. Another issue pertains to the uncertainty of solute recovery across the dialysis fiber in vivo, relative to the in vitro measurement of solute recovery [62]. The recovery of a therapeutic antibody was 3% across the fiber, but this recovery varied 50-fold depending on changing experimental conditions [57].

### 3.3. Log BB Ratio

The log of the BB ratio, which is the brain:blood ratio, at a defined time point, e.g., 60 min after drug administration, is used as a measure of BBB transport of small molecule drugs [63]. However, the BB ratio is a volume of distribution (VD), and is largely determined by differential binding of small molecule drugs to brain proteins [64]. Two drugs may have similar BBB permeability, but differ widely in log BB ratios, if there is differential binding of the drugs by brain tissue. Binding of small molecule drugs by cytoplasmic proteins in brain, including AD brain, may be assessed by equilibrium dialysis of brain homogenates [65]. For large molecule drugs, it is important to correct the BB ratio, i.e., the brain VD, with the brain blood volume (Vo), as discussed below for computation of the BBB permeability-surface area (PS) product.

### 3.4. BBB PS Product

The best measure of BBB permeability of a drug is the PS product [64], which has units of mL/min/gram brain, and is the product of the BBB permeability coefficient, P, with units of cm/min, and the brain capillary surface area, S, with units of cm^2^/g brain. The PS product is defined as,

PS product = [(VD − Vo) ∗ Cp(T)]/[AUC(T)],

where VD is the brain/plasma ratio, in uL/g at a defined time (T) after injection, Vo is the brain plasma volume, in uL/gram, Cp(T) is the plasma drug concentration at time, T, and AUC(T) is the area under the plasma concentration curve from the time of injection to time, T, when the uptake period is terminated [64]. The PS product for small molecule drugs should be measured at short time intervals following drug injection, so as to eliminate drug efflux from brain during the experimental time period. For a drug that rapidly crosses the BBB, this experimental time period should be brief, e.g., 15 s. Such brief experimental time periods also minimize artifacts caused by rapid metabolism of the drug. It may be difficult to determine the plasma AUC over such short time period. In this case, the external organ method can be used, where an arterial catheter is implanted and connected to a syringe withdrawal pump, and the average plasma concentration in the syringe over the 15 s period is a measure of the plasma AUC [66]. For large molecules, there may be minimal efflux in time periods of 1–2 h, and the plasma AUC can be determined by standard pharmacokinetics methods. Drug concentrations in brain and plasma can be determined by radio-isotopic methods, mass spectrometry, HPLC, or ELISA. If there is minimal penetration of the BBB by the drug, then the VD and Vo values will not be significantly different, and the PS product is zero, as shown in the above equation for PS. If careful measurement of the brain plasma volume, Vo, are not made, then the PS value will be artifactually >0, owing to entrapment of the drug within the blood volume of brain.

### 3.5. Measurement of Free Drug in Brain

Drug receptors in brain are generally targeted by free drug in brain tissue in vivo. Attempts have been made to estimate free drug in brain with equilibrium dialysis of brain homogenates in vitro [65]. However, it is difficult to predict free drug in brain in vivo with the use of in vitro homogenates. This is because the input, or forcing function, that exists in vivo, which is the exchangeable drug in plasma that is continuously entering into the brain compartment [67], is non-existent with in vitro homogenates of brain. The free drug in brain in vivo (a) is directly related to the input function, i.e., the plasma exchangeable (transportable) drug in plasma, (b) is inversely related to the rate of drug metabolism in brain, and (c) is independent of brain tissue binding of the drug [68]. Whereas brain tissue binding of drug does not affect the free drug in brain in vivo, tissue binding of drug in brain is directly proportional to the total drug concentration in brain [67,68], as reflected in the brain VD or log BB. The measurement of the exchangeable drug in plasma with in vitro equilibrium dialysis of plasma may under-estimate the plasma exchangeable drug in vivo if there is enhanced dissociation of drug from drug binding plasma proteins within the brain capillary endothelial compartment in vivo [68].

## 4. Small Molecule Drugs for Alzheimer’s Disease

### 4.1. Mechanism of BBB Transport of Small Molecules

Small molecule drugs are generally believed to cross the BBB without a delivery system, thus leading to a development path much shorter than biologic drugs. However, apart from affective disorders, <2% of all small molecule drugs are active in the brain [7]. Of >7000 drugs in the Comprehensive Medicinal Chemistry database, only 5% of all drugs are active in the CNS, and these drugs are only active for affective disorders and insomnia [69]. In another survey, 12% of all drugs were active in the CNS, but this number dropped to 1% if affective disorders were excluded [70]. The reason so few small molecule drugs are active in CNS, apart from affective disorders, relates to the molecular properties of small molecules that enable BBB passage. First, if the MW of the drug exceeds 400 Da, BBB transport is minimal [71]. Second, if the polar functional groups on the drug form more than 7 hydrogen bonds [72], then the drug is too polar to cross the BBB. If the polar surface area (PSA) of the drug exceeds 80 Å^2^, which corresponds to a MW of 400 Da, then BBB transport is minimal [71]. BBB transfer decreases exponentially when the PSA increases from 52 Å^2^, which corresponds to a MW of 300 Da, to a PSA of 105 Å^2^, which corresponds to a MW of 450 Da [71]. The number of brain disorders that responds to small molecules with a MW < 400 Da, and a hydrogen bond number of <7, is limited, and is restricted to affective disorders, pain, insomnia, and epilepsy [72]. The only small molecules approved for AD, as discussed below, are the acetylcholinesterase inhibitors (ACEI), and these drugs have a MW ranging from 179 Da to 380 Da, and form ≤5 hydrogen bonds with solvent water [73].

**Figure 2:**
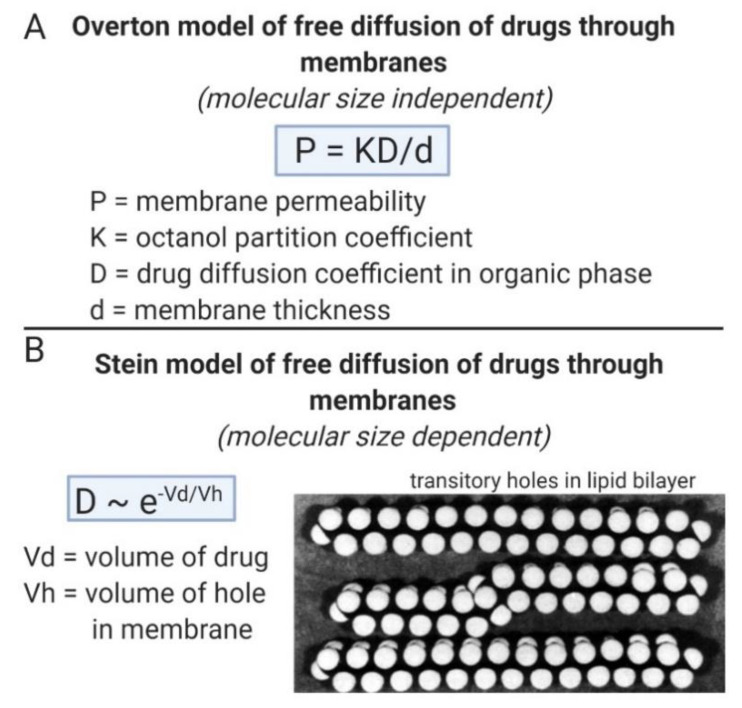
(**A**) The Overton model of solute free diffusion through biological membranes defines the membrane permeability as a property of lipid solubility, diffusion coefficient, and membrane thickness, and is independent of the molecular weight (MW) of the solute [74]. (**B**) In the Stein model [74] of solute diffusion through biological membranes, the drug diffusion coefficient is dependent on MW, in that MW is generally proportional to the molecular volume (Vd) of the drug. The drug diffusion coefficient decreases exponentially as the Vd increases relative to the volume of membrane holes (Vh) [74]. The membrane holes through which the solute penetrates the membrane are formed by the transitory kinking of fatty acyl side chains of membrane phospholipids; the membrane model is adapted from Trauble [75] with permission. Image created with Biorender.com.

The MW threshold for BBB transfer of small molecules is the limiting factor in the discovery of new small molecule drugs for neurodegenerative disease, because drugs discovered from receptor-based high throughput screening (HTS) invariably have a MW > 500 Da [70]. The mechanism of small molecule transport across phospholipid membranes, which gives rise to the MW threshold, is shown in Figure 2. In the classical Overton model of solute free diffusion through membranes [74], the permeability coefficient (P) is a function of the lipid partition coefficient (K), e.g., the K in a model solvent such as 1-octanol, the drug diffusion coefficient (D), and the thickness of the membrane (d), as described in Figure 2A. The Overton model is MW independent [74]. The Stein model [74] of solute free diffusion through membranes defines D as a function of the volume of the drug (Vd), relative to the volume of membrane holes (Vh), as shown in Figure 2B. In this model, the diffusion of the solute through the membrane is viewed as a process where the drug jumps from hole to hole within the membrane until diffusion through the membrane is completed. The transitory holes within the phospholipid bilayer are formed by transient kinking of the free fatty acyl side chains within the membrane, as postulated by Trauble [75], and shown in Figure 2B. The threshold of PSA of the drug that limits drug diffusion through the membrane is defined by the volume of the transitory holes within the membrane. The presence of a MW threshold for small molecule transport through the BBB [76], was first discussed by Levin [77].

### 4.2. Acetylcholinesterase Inhibitors for Alzheimer’s Disease

The first and largely the only class of small molecules approved for the treatment of AD, are the acetylcholinesterase inhibitors (ACEI). The ACEIs, donepezil, rivastigmine, tacrin, and galantamine, all have a MW of 198–380 Da and form ≤5 hydrogen bonds [73]. Tacrin was FDA approved for AD in 1993, and has since been discontinued. The last drug of any class that was approved by the FDA was memantine, which was approved in 2003. Memantine is a low affinity blocker of N-methyl D-aspartate (NMDA) glutamate receptors. Memantine has a MW of 179 Da and forms only three hydrogen bonds, and is recommended when the patient does not respond to an ACEI. However, these FDA approved small molecule drugs have had a questionable benefit in the treatment of AD [78]. Consequently, small molecule drug discovery for AD has focused on inhibitors of secretase enzymes that give rise to the Abeta peptide in AD.

### 4.3. Secretase Inhibitors for Alzheimer’s Disease

The Abeta peptide of AD is derived from the enzymatic processing of the amyloid peptide precursor (APP). A 99-amino acid peptide, CT99, also called C-terminal fragment (CTF), is cleaved near the carboxyl terminus of APP by the beta site APP cleaving enzyme type 1 (BACE1), also known as beta secretase [79]. The CT99 peptide is cleaved to form the 43 amino acid Abeta peptide by gamma secretase (GS) [80]. Both small molecule BACE1 inhibitors (BACE1I), and GS inhibitors (GSI), have been developed [73], and several have entered clinical trials for AD, as listed in Table 1. 

None of these BACE1Is, or GSIs, have been approved by the FDA, and the majority of the clinical trials have been discontinued (Table 1). These drugs have an average MW of 422 Da, and form, on average, 6 hydrogen bonds (N), and these molecular properties predict minimal BBB penetration. However, several BACE1 or GSI have properties that enable BBB transport, and lower brain Abeta in mouse models of AD [82]. Nevertheless, these drugs have failed in clinical trials owing to multiple factors, including toxicity, or lack of BACE1 specificity [82]. An alternative class of drugs under clinical investigation for AD are small molecule blockers of Abeta fibril formation.

### 4.4. Abeta Fibril Inhibitors

Potent inhibitors of the formation of Abeta fibrils or plaques are therapeutic antibodies, as described below. A small molecule that blocks the formation of Abeta dimers is homotaurine (tramiprosate) [83], which is a highly polar molecule, 3-amino-1-propanesulfonic acid, that, in the absence of carrier mediated transport, would be expected to have minimal BBB transport. A clinical trial of homotaurine for AD did not result in an improvement in cognitive decline [84]. ALZ-801, which is in clinical trials for AD (Table 1), is a homotaurine prodrug, wherein the primary amino terminus is converted to an aliphatic amide group [85]. Such a modification would reduce the hydrogen bonding by 1, but the main problem with ALZ-801, with respect to BBB transport, is the presence of the free sulfonic acid moiety. The presence of a sulfonic acid group reduces lipid solubility by three orders of magnitude, and to an extent greater than any other functional group [86]. Consequently, the brain uptake of MK-801 is only marginally superior to the brain uptake of tramiprosate [85]. Cromolyn reduces soluble Abeta peptide, and is in clinical trials for AD (Table 1). Cromolyn has a high MW of 468 Da, and is highly polar and forms 10 hydrogen bonds. These properties would predict minimal, if any, BBB transport. ELND005 is an inositol derivative that blocks Abeta oligomers and is in clinical trials for AD (Table 1). However, this molecule is highly polar, forming 12 hydrogen bonds, and is predicted to have minimal BBB penetration.

### 4.5. Summary of Small Molecule Drug Development for Alzheimer’s Disease

No small molecule drug has been FDA approved for AD since 2003 [6], despite a global effort by the pharmaceutical industry to develop small molecule inhibitors of Abeta peptide formation or oligomerization. The problem with the small molecule approach to CNS drug discovery, particularly receptor-based HTS drug discovery, is that the drugs invariably have a MW > 400 Da and/or form >7 hydrogen bonds [70], and these properties lead to minimal BBB transport. Given the difficulty in developing small molecule drugs for AD, the pharmaceutical industry has made a major effort in the drug development of biologics for AD, mainly therapeutic antibodies, and these agents are discussed below.

## 5. Biologics for Alzheimer’s Disease

### 5.1. Amyloid-Beta Therapeutic Antibodies for Alzheimer’s Disease

The dementia of AD correlates with the deposition in brain of amyloid-beta plaques [2,3] and tau neurofibrillary tangles [87,88]. The intra-cerebral injection of monoclonal antibodies (MAb), particularly an MAb against the amino terminus of the Abeta peptide, results in plaque disaggregation [89]. The anti-Abeta antibody (AAA) was administered by intra-cerebral injection, because AAAs do not cross the BBB [90]. Subsequent studies in AD transgenic mice showed the systemic administration of large doses of the AAA reduced amyloid plaque in brain, owing to AAA entry into brain parenchyma [91]. However, this treatment also caused cerebral microhemorrhage [26], which results in BBB disruption, a mechanism by which the AAA may penetrate the BBB. The first AAA to enter clinical trials in AD was bapineuzumab, a humanized version of the mouse 3D6 MAb [92]. In the design of the bapineuzumab clinical trial, it was assumed that this AAA crosses the BBB, but preclinical research in mice showed the brain uptake of bapineuzumab was only 0.07% of injected dose (ID)/gram brain [93], a level of brain uptake consistent with entrapment in the brain blood volume. The administration of high doses of bapineuzumab to patients with AD caused vasogenic edema and BBB disruption, based on the findings of ARIA-E on MRI [94]. This vasogenic edema was the clinical correlate of the cerebral microhemorrhage in AD transgenic mice administered high doses of an AAA [26]. Subsequently, two phase 3 clinical trials of bapineuzumab in AD failed [95]. Despite the negative bapineuzumab trial, the industry developed over a dozen different AAAs, all of which entered into clinical trials for AD (Table 2). In most cases, the clinical trial was terminated without a successful outcome (Table 2). With one exception, the AAAs listed in Table 2 are expected to have a difficult path to FDA approval, because the AAA does not cross the non-disrupted BBB. The one exception in Table 2 is RO7126209, which is a bispecific antibody (BSA) formed by fusion of gantenerumab, an AAA that failed in clinical trials [96], to a single chain Fab antibody against the human transferrin receptor (TfR) [97]. This BSA for AD follows the original description of a BSA formed by fusion of a single chain Fv (ScFv) antibody directed at the Abeta peptide of AD to the carboxyl terminus of either a MAb against the human insulin receptor (HIR), designated HIRMAb [90], or to a MAb against the mouse TfR, designated the TfRMAb [98]. The TfRMAb or HIRMAb domain of these BSAs is designed to trigger receptor-mediated transport across the BBB via the endogenous TfR or HIR expressed on the human BBB [8]. The results of the phase 1 trial of RO7126209 are not available, and whether this BSA will progress to a phase 2/3 clinical trial in AD remains to be reported.

AAAs have entered clinical trials for AD without sufficient evidence that the drug actually crosses the BBB. Aducanumab was said to cross the BBB [22], but an analysis of this data shows this antibody is likely confined to the brain blood volume [25]. In clinical trials, aducanumab reduces amyloid plaque in AD [22], which is indicative of antibody penetration into the brain. However, there is a linear relationship between the dose of aducanumab that causes plaque reduction and the dose that causes ARIA-E [22], which suggests that the mechanism of aducanumab entry into brain is BBB disruption. Clinical trials of aducanumab in AD fail to show a consistent effect on cognitive decline, although a post-hoc analysis of the data is ongoing [99]. Solanezumab, which failed in AD clinical trials, is a AAA that was said to have a high level of target engagement in AD [100]. The assessment of this target engagement was based on measurements of Abeta in CSF, a volume of brain that is accessible to therapeutic antibodies, owing to the leakiness of the choroid plexus (Figure 1). An inexplicable aspect of AAA drug development in AD is the failure to focus on the role of the BBB in clinical trial results. In a recent review of three clinical trial failures of therapeutic AAAs for AD, aducanumab, crenezumab, and solanezumab, the role of the BBB is not considered [6].

### 5.2. Tau Therapeutic Antibodies for Alzheimer’s Disease

The neurofibrillary tangles in AD are caused by the aggregation of the Tau protein, an intracellular microtubule-related protein [101]. Tau monomers form fibrils and then aggregates similar to the Abeta peptide. Tau aggregates are primarily intracellular, but Tau is also secreted to the brain extracellular (EC) space as fragments that can lead to transcellular seeding of Tau aggregates [102]. Some anti-tau antibodies (ATA), e.g., gosuranemab [58], bind to the amino terminal (NT) region of Tau (Table 3), whereas other ATAs bind hyper-phosphorylated forms of Tau (Table 3). Gosuranemab is said to engage the target within brain based on evidence derived from either CSF or microdialysis measurements, although no brain, or brain/plasma ratios, of the antibody were reported [58]. As discussed above, both CSF and microdialysis studies may not predict ATA penetration of the BBB. Antibody penetration into CSF is expected based on the leakiness of the choroid plexus (Figure 1). Antibody penetration into the ISF region around a microdialysis fiber implant is expected based on the BBB disruption associated with the intra-cerebral insertion of the dialysis fiber. ATAs were found not to cross the intact BBB, which provided the rationale for increasing brain uptake of the ATA via BBB disruption caused by the IV administration of microbubbles in parallel with ultrasonic irradiation of the brain [103].

### 5.3. TREM2 Therapeutic Antibodies for Alzheimer’s Disease

Triggering receptor expressed on myeloid cells 2 (TREM2) is produced in the CNS in microglial cells and the loss of the anti-inflammatory properties of this receptor is associated with increased tau pathology, and in older mice, increased amyloid plaque in preclinical models of AD [104,105]. AD is a state of chronic neuro-inflammation [5]. Therefore, a therapeutic antibody with TREM2 agonist properties, AL002 [23], has been developed as a new treatment for AD, and is the first TREM2 antibody to enter clinical trials in AD (Table 3). Evidence that this therapeutic antibody crosses the intact BBB was produced by measurement of brain and plasma antibody concentrations in mice administered 5–60 mg/kg of the antibody by intra-peritoneal injection [23]. The brain was cleared of plasma by intra-cardiac saline perfusion prior to sampling of brain for antibody levels. The brain uptake was low at all doses, 0.11% ID/gram [23], which is a level of brain uptake similar to bapineuzumab [93]. The brain/plasma ratio of the antibody, which is the brain volume of distribution (VD), was 2 uL/gram [23], which is comparable to the brain VD of aducanumab [22], which is a fraction of the brain blood volume. This low level of brain uptake of this antibody may represent entrapment in the brain blood volume owing to incomplete washout of this compartment [25].

### 5.4. Neurotrophins for Alzheimer’s Disease

Biologic drug development in AD has focused on reduction of amyloid plaque or NFTs. However, the dementia of AD is linked to the dystrophic neurites produced in brain following the deposition of amyloid plaque or NFTs [40]. The neurotrophin, nerve growth factor (NGF), was proposed as a treatment for AD over 30 years ago [37]. Apart from NGF, there are over 15 other neurotrophins that are potential treatments of AD [40]. The problem is that neurotrophins do not cross the BBB, and past clinical neurotrophin trials in neurodegeneration delivered the neurotrophin to brain via either direct injection into CSF [41], or into brain tissue [47]. These clinical trials failed owing to the limitations of diffusion of the agent within the brain. Adequate delivery of neurotrophins to brain is possible via the transvascular route across the BBB following the re-engineering of the neurotrophin as a BBB penetrating IgG fusion protein, as discussed below.

### 5.5. Summary of Biologics Drug Development for Alzheimer’s Disease

Biologic drugs, therapeutic antibodies, decoy receptors, neurotrophins, are large molecule drugs that do not cross the intact BBB. Therefore, for such agents to fail repeatedly in AD clinical trials is not unexpected, since the BBB is intact in AD. Although Abeta oligomers alter brain endothelial tight junction proteins in cell culture [106], the BBB in vivo in AD is intact, based on PET [107], computed tomography [108], and MRI [109]. Given the enormity of societal costs of AD [1], and that there have been no new drugs approved for AD since 2003 [6] despite a global effort on the part of the pharmaceutical industry, it is time to take a new approach to AD drug development. In this new approach, BBB drug delivery technology is developed in parallel with CNS drug discovery for AD, so that new drug candidates are re-engineered to enable BBB transport and penetration of the brain from blood. This is possible for biologics with BBB Trojan horse technology. A BBB Trojan horse is a peptide, or a peptidomimetic MAb, that binds an endogenous receptor-mediated transport (RMT) system on the BBB, such as the endogenous BBB insulin receptor (IR) or TfR [110]. The endogenous BBB RMT system transports genetically engineered fusion proteins where the therapeutic antibody, decoy receptor, or neurotrophin, is fused to the BBB-penetrating IgG Trojan horse [8].

## 6. Re-Engineering Biologics as BBB-Penetrating IgG Fusion Proteins for Alzheimer’s Disease

### 6.1. Receptor-Specific BBB Trojan Horses for Brain Drug Delivery of Biologics

L-DOPA is a neuroactive drug for PD, because this neutral amino acid drug penetrates the BBB via the large neutral amino acid carrier-mediated transport (CMT) system on the BBB [111], which is the type 1 large neutral amino acid transporter (LAT1) [112]. Many other CMT systems are expressed on the BBB for other classes of nutrients, vitamins, or hormones, and these endogenous CMT systems are potential portals of entry to the brain of small molecule CNS drugs that have been modified to enable transport across the BBB on the CMT systems [113]. Similarly, receptor-mediated transport (RMT) systems are expressed within the BBB to mediate the transport of endogenous peptides, such as insulin, transferrin (Tf), leptin, or the insulin-like growth factors (IGFs) [8]. The BBB RMT systems also transport peptidomimetic MAbs that bind exofacial epitopes on the BBB receptor, and this binding allows the antibody to piggyback across the BBB on the endogenous RMT system. The RMT of receptor specific antibodies across the BBB has been known for over 25 years, and was shown for a TfRMAb in the rat [114], and a HIRMAb in the Rhesus monkey [115]. These BBB-specific antibodies can act as a molecular Trojan horse for delivery of biologics, which alone, do not cross the BBB.

**Figure 3:**
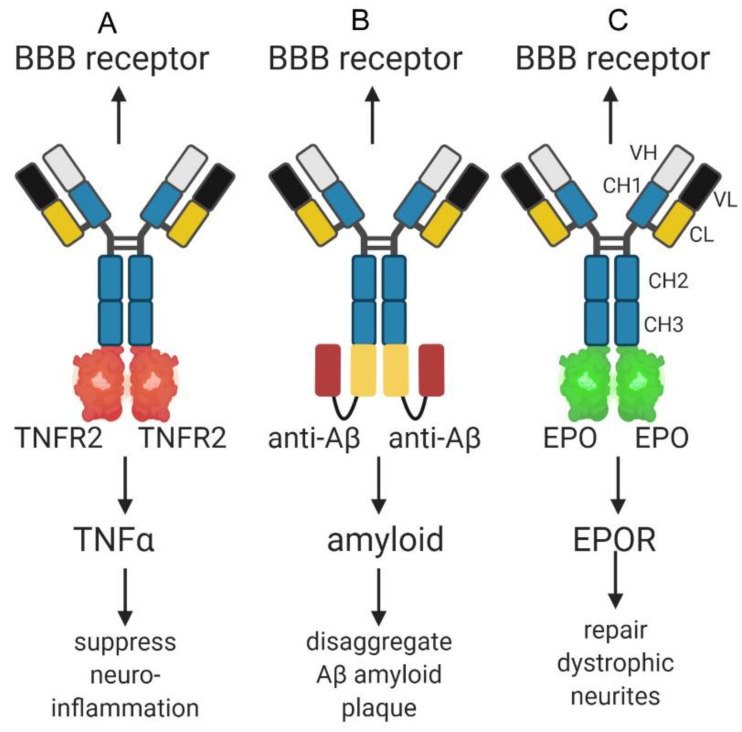
(**A**) IgG-TNFR2 fusion protein formed by fusion of TNFR2 extracellular domain (ECD) to carboxyl terminus of the heavy chain of a monoclonal antibody (MAb) against the BBB insulin receptor (IR) or transferrin receptor (TfR). The fusion protein both binds the receptor on the BBB, to enable entry into brain, and sequesters TNFα in brain to suppress neuro-inflammation. (**B**) Bispecific antibody formed by fusion of a single chain Fv anti-Abeta antibody to the carboxyl terminus of the heavy chain of a MAb against the BBB IR or TfR. The fusion protein both binds the receptor on the BBB to cause penetration of the brain, and disaggregates amyloid plaque in brain behind the BBB. (**C**) IgG-erythropoietin (EPO) fusion protein formed by fusion of EPO to the carboxyl terminus of the heavy chain of a MAb against the BBB IR or TfR. The fusion protein both binds a receptor on the BBB, to trigger entry into brain, and binds the EPO receptor (EPOR) in brain to induce neuroprotection. Image created with Biorender.com.

So as to enable BBB delivery of biologics, in early work the drug was conjugated to the TfRMAb or HIRMAb with avidin-biotin technology. A monobiotinylated peptide, e.g., vasoactive intestinal peptide (VIP), or the Abeta peptide, were bound to a chemical conjugate of streptavidin and the TfRMAb or HIRMAb [116,117]. Subsequently, IgG fusion proteins were genetically engineered, where the biologic was fused to the TfRMAb or HIRMAb [110]. All classes of biologics, including enzymes, decoy receptors, neurotrophins, and therapeutic antibodies were fused to a TfRMAb or a HIRMAb Trojan horse [110], and model IgG fusion proteins are shown in Figure 3 for a decoy receptor, a therapeutic antibody, and a neurotrophin. In the case of a decoy receptor, the extracellular domain (ECD) of the type 2 tumor necrosis factor receptor (TNFR2) was fused to the transporting IgG [118]. This TNFR2 ECD is the active moiety in etanercept, which is a TNFR2-Fc fusion protein. A model therapeutic antibody against the Abeta peptide of AD was first re-engineered as a single chain Fv (ScFv) antibody, and this ScFv antibody was then fused to the transporting IgG [90]. A model neurotrophin, erythropoietin (EPO), was fused to the transporting IgG [119]. Alone, etanercept, the AAA, or EPO, do not cross the primate BBB [90,118,119]. However, high brain uptake, 1–3% ID/brain, was observed in the primate for the HIRMAb-TNFR2 fusion protein, the HIRMAb-AAA fusion protein, and the HIRMAb-EPO fusion protein [90,118,119]. This level of brain uptake in the Rhesus monkey is comparable to the brain uptake of a lipid soluble small molecule, fallypride, which is 2–3% ID/brain in the primate [120].

### 6.2. IgG-Decoy Receptor Fusion Proteins for Alzheimer’s Disease

Etanercept by SQ injection has been tested in clinical trials for AD without success [121]. This was the expected result, since etanercept does not cross the BBB [118]. The absence of BBB transport of etanercept was subsequently confirmed by single photon emission computed tomography (SPECT) after [^125^I]-etanercept administration by peri-spinal IV injection [122]. A BBB-penetrating form of etanercept could be an important new drug for the treatment of AD, since tumor necrosis factor (TNF)α plays a role in the chronic neuro-inflammation of AD [123], as well as other neurodegenerative diseases, such as PD [124]. A TfRMAb-TNFR2 fusion protein is therapeutic following systemic administration in a transgenic mouse model of AD [125]. AD mice were treated thrice-weekly for 12 weeks by intra-peritoneal (IP) injection of 3 mg/kg of the TfRMAb-TNFR2 fusion protein, and this treatment caused a reduction in brain amyloid plaque, a decrease in brain immunoreactive intercellular adhesion molecule 1 (ICAM1), a marker of neuroinflammation, and an increase in recognition memory in the AD mice [125]. Chronic treatment of the AD mice produced only a low titer anti-drug antibody (ADA) response [125]. The re-engineering of biologic TNF inhibitors (TNFI), such as etanercept or adalimumab, to enable transport across the BBB could be new treatments to suppress the neuro-inflammation in AD.

### 6.3. Bispecific Antibodies for Alzheimer’s Disease

When the BBB Trojan horse is an IgG, and the biologic for AD is a therapeutic antibody, then the problem is engineering a bispecific antibody (BSA), where the Trojan horse antibody is genetically fused to the therapeutic antibody. So as to retain high affinity for both the CNS target and for the BBB receptor, a tetravalent BSA was engineered (Figure 3B), where a ScFv against the Abeta peptide was fused to the carboxyl terminus of each heavy chain of a TfRMAb [98], for mouse studies, or to a HIRMAb [90], for primate investigations. Chronic treatment of a transgenic AD mouse with 5 mg/kg of the TfRMAb-ScFv fusion protein by subcutaneous (SQ) administration for 12 weeks caused a 60% reduction in brain amyloid plaques without cerebral micro-hemorrhage [98]. Chronic treatment of the mice produced only a low titer ADA response. In an alternative format, a tetravalent BSA was engineered by fusion of a ScFv derived from the 8D3 MAb against the mouse TfR to the carboxyl terminus of each light chain of the mAb158 anti-Abeta antibody [126]. The mAb158 is the mouse homologue of the BAN2401 antibody (Table 2). Following IV injection in mice, the brain uptake of the mAb158 antibody was low, 0.13% ID/gram [29], which is comparable to the brain uptake of other anti-Abeta antibodies, bapineuzumab [93] or aducanumab [22]. In contrast, the brain uptake of the mAb158-derived BSA targeting the TfR on the BBB was 1.0% ID/gram brain [29]. A monovalent BSA was engineered with knob-in-hole technology, where one monovalent arm of the BSA was directed against the TfR on the BBB, and the other monovalent arm of the BSA was a directed against BACE1 [127]. A monovalent/bivalent BSA was engineered by fusion of a single chain Fab antibody against the TfR on the BBB to the carboxyl terminus of a single heavy chain of mAb31 [97], the mouse homologue of gantenerumab (Table 2). This BSA was the precursor to RO7126209 (Table 2), which is the first BBB penetrating BSA to enter clinical trials. A monovalent/bivalent BSA was also engineered with knob-in-hole technology where the amino-terminal domain of the BSA was bivalent and directed against BACE1, and the carboxyl terminal domain was monovalent for the human TfR [128]. The latter binding site was created in a single CH3 region of the Fc domain of the BSA via the mutation of designated amino acids. This approach yields a TfR-directed antibody of very low affinity with a dissociation constant (KD) of 120 nM for the human TfR, and a KD of 1900 nM for the cynomolgous TfR [128].

### 6.4. IgG-Neurotrophin Fusion Proteins for Alzheimer’s Disease

The EPO receptor (EPOR) is expressed in brain, and EPO is a potential neuroprotective neurotrophin for AD and other CNS disorders [129]. The neuroprotection of intravenous EPO in stroke was examined, but this clinical trial failed [130], because (a) EPO does not cross the intact BBB [119], and (b) the BBB is intact in the early hours after stroke when neuroprotection is still possible [131]. The basis for the EPO stroke trial was the finding of EPO distribution into CSF [132]. However, as discussed above, CSF is not a useful index of drug penetration into brain parenchyma across the BBB, owing to the leakiness of the choroid plexus. When BBB transport of EPO is measured directly, this neurotrophin does not cross the BBB [119]. Another problem with EPO as a neuroprotective agent is the potential hematopoietic effect of chronic EPO treatment. The hematopoietic effect of EPO is inversely related to the plasma clearance rate [133]. Both issues, lack of BBB transport and hematopoietic effect, can be addressed by the re-engineering of EPO as an IgG fusion protein with a BBB molecular Trojan horse [119]. Following fusion of EPO to the HIRMAb, the brain uptake of EPO is 2% ID/brain in the Rhesus monkey, which is a level of brain uptake comparable to small molecules [119]. Chronic treatment of mice with a TfRMAb-EPO fusion protein produced only a minor increase in hematocrit [134], which was attributed to the 15-fold increase in plasma clearance of the IgG-EPO fusion protein, as compared to EPO alone [119]. A TfRMAb-EPO fusion protein was administered to AD transgenic mice by thrice-weekly IP injections at 3 mg/kg for 8 weeks. This treatment caused a reduction in synaptic loss, which correlated with decrease in microglial activation, and an increase in spatial memory [135]. The re-engineering of neurotrophins, such as EPO, NGF, or multiple other neuroprotective neurotrophins, as BBB penetrating IgG-neutrophin fusion proteins, could lead to new treatments to repair dystrophic neurites in AD.

### 6.5. Combination Biologics Therapy of Alzheimer’s Disease

**Figure 4:**
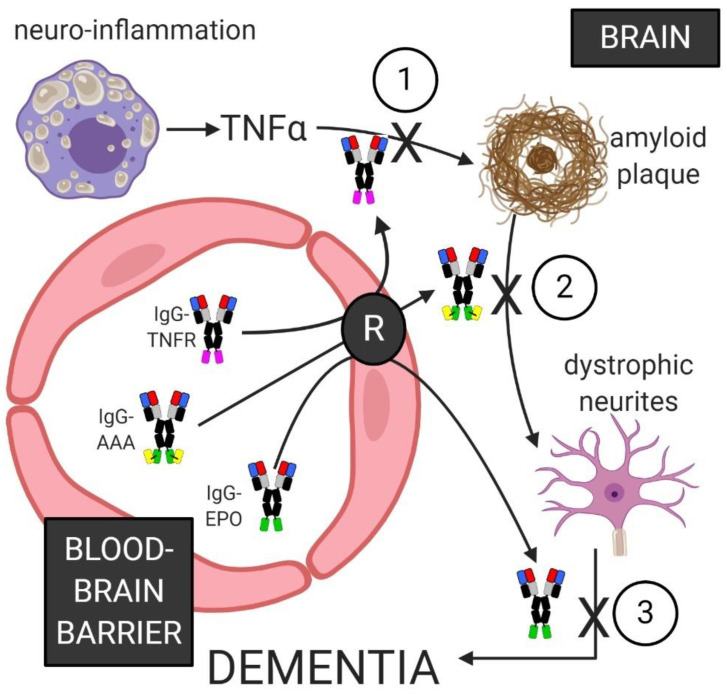
Blood-borne IgG fusion proteins penetrate the BBB via transport on a brain capillary endothelial receptor (R). An IgG-TNFR fusion protein sequesters TNFα to suppress neuro-inflammation. An IgG-AAA fusion protein disaggregates amyloid-beta plaque. An IgG-EPO fusion protein activates the neuronal EPOR to induce repair of dystrophic neurites, which leads to resolution of the dementia of AD. TNFR: tumor necrosis factor receptor; AAA: anti-Abeta antibody; EPO: erythropoietin. Image created with Biorender.com.

The pathogenesis of the dementia of AD may be the result of a cascade of cellular processes in the brain, which involve neuro-inflammation, formation of amyloid and Tau plaques, and neurite loss and dystrophy, as illustrated in Figure 4. A therapy that only reduces amyloid plaque in AD brain may not improve clinical dementia scores, if the dystrophic neurites are not repaired, e.g., with neurotrophin therapy. The reduction of amyloid plaque and the repair of dystrophic neurites may not result in a lasting therapeutic response in AD if the chronic neuro-inflammation is not abated. Therapies have existed for years to treat each of the cellular processes shown in Figure 4: neuro-inflammation leading to amyloid plaque, which then leads to dystrophic neurites, which leads to the dementia of AD. Biologic TNFIs, such as entanercept, or adalimumab, both suppress TNFα in the peripheral organs, but these TNFIs cannot be used in AD, because the TNFIs do not cross the BBB. Therapeutic antibodies against either Abeta or Tau are widely prevalent, and many have entered clinical trials (Table 2 and Table 3). However, such therapeutic antibodies do not cross the BBB, and the BBB is intact in AD [107,108,109]. Neurotrophins, e.g., NGF, were proposed for AD nearly 35 years ago [37], but neurotrophins do not cross the BBB. As discussed above in the small molecule section, a small molecule neurotrophin mimetic that has high affinity for the target receptor will unlikely have the molecular characteristics, MW < 400 Da and low hydrogen bonding, that enable small molecule transport across the BBB. Target-specific small molecules invariably have a MW > 500 Da [70]. However, solutions to the BBB problem are now available for biologics. A TNFI, such as the TNFR2 ECD, can be re-engineered as an IgG-TNFR2 fusion protein that crosses the BBB. A therapeutic antibody against Abeta, Tau, or TREM2 can be re-engineered as a BBB-penetrating BSA. A neurotrophin can be RE-engineered as an IgG-neurotrophin fusion protein that is BBB penetrating and neuroprotective in AD. Such combination therapy with biologics that cross the BBB is possible with BBB Trojan horse technology. Such BBB-penetrating biologics can interfere in the different parts of the pathologic cascade that leads to the dementia of AD (Figure 4).

### 6.6. Safety of BBB Trojan Horse Fusion Proteins

Safety issues could arise following the administration of BBB Trojan horse IgG fusion proteins due to either the therapeutic domain or the Trojan horse domain of the fusion protein. The acute administration of a TfRMAb results in a marked decrease in blood reticulocytes within 24 h [136]. This affect arises from an effector function of the antibody, and interaction of the carbohydrate domain within the Fc region of the TfRMAb and the gamma Fc receptor (FcR). However, this effector function effect on reticulocytes is short-lived, as reticulocyte counts in blood return to normal by at least 7 days after a single administration of a TfRMAb, and reticulocyte counts are normal following 4 weeks of chronic administration of a TfRMAb in mice [137]. A TfRMAb that both crosses the BBB, and activates effector function at Fc receptors, may have effects within the brain, since astrocytes and microglia express the FcR [138]. The effector function of the TfRMAb can be eliminated by production of aglycosylated forms of the antibody via mutation of the asparagine (Asn) residue within the Fc region of the antibody [127]. Mutation of the Asn at position 292 of the antibody to a glycine residue, which is designated the N292G mutation, causes accelerated plasma clearance of a TfRMAb-EPO fusion protein in mice [139]. Chronic administration of a TfRMAb alone also causes an acceleration of plasma clearance of the antibody in both monkeys [138] and mice [137]. In contrast, chronic administration of a TfRMAb-GDNF fusion protein does not alter the plasma clearance of the fusion protein in mice [140]. Therefore, the distribution in vivo of a TfRMAb fusion protein may not parallel that of the TfRMAb alone.

The first BBB Trojan horse fusion protein tested in human clinical trials was a fusion protein of iduronidase (IDUA), the lysosomal enzyme mutated in Mucopolysaccharidosis Type I (MPSI), and a HIRMAb, and this fusion protein is alternatively designated as the HIRMAb-IDUA fusion protein or valanafusp alpha [141]. IV infusion of high doses of the HIRMAb-IDUA fusion protein in saline causes hypoglycemia in primates [142]. However, the hypoglycemia is eliminated by infusion of the HIRMAb-IDUA fusion protein in saline with dextrose [142]. Treatment of pediatric subjects with MPSI for 52 weeks by weekly IV infusions of 1–3 mg/kg of valanafusp alpha in 5% dextrose/saline produced an incidence of either mild reversible hypoglycemia, or infusion related reactions, of ≤2%, and no effector function side effects [141]. Treatment of primates with SQ injections of 3–30 mg/kg of the HIRMAb alone produces no hypoglycemia [143]. Long-term treatment of monkeys with 3–30 mg/kg of either the HIRMAb-IDUA fusion protein for 6 months by weekly IV infusion [144], or with 3–30 mg/kg of the HIRMAb alone for 3 weeks by repeated SQ injections [143], has no effect on the plasma clearance of either the antibody alone or the fusion protein. Long term treatment with the HIRMAb-IDUA fusion protein has no neuropathologic effects in primates [144].

In summary, the therapeutic index of BBB Trojan horse-biologic fusion proteins is a function of both the therapeutic domain and the IgG transporting domain. Efficacy in brain is a function of both the BBB transporting activity of the Trojan horse domain, as well as the intrinsic potency of the therapeutic domain. Safety issues are also a function of both the IgG transporting domain and the therapeutic domain of the fusion protein.

## 7. Conclusions

There has been a striking failure in global drug development for AD, as there has not been a new drug approved for AD since 2003 [6]. While the difficulty in finding new treatments that yield positive clinical trials in AD is indisputable, the history of AD drug development is inexplicable in the context of the blood–brain barrier. Given that 98% of all small molecule drugs, and ~100% of all biologics, do not cross the BBB [7], how could it be that the BBB is systematically ignored in AD drug development? A 2020 EU/US Clinical Trials for Alzheimer’s Disease (CTAD) Task Force reviews current AD drug development and never mentions the BBB [145]. A comprehensive 2019 review of 132 drugs in AD clinical trials never discusses the role of the BBB in AD drug development [33]. A 2020 review of failed clinical trials in AD with therapeutic antibodies does not mention the BBB [6]. It is not realistic to believe that new treatments of AD are forthcoming if the drugs under investigation do not cross the BBB. The first step in the design of new drugs for AD that penetrate the BBB is the incorporation of BBB drug delivery technology in AD drug development.

## Figures and Tables

**Table 1:** Small molecules targeting Abeta in clinical trials for Alzheimer’s disease.

Drug	Mechanism	MW	Polarity	Clinical Trial
ALZ-801	Block Abeta dimers	238	Sulfonic acid	Completed; NCT04157712
cromolyn	Reduces soluble Abeta peptide	468	N = 10	Phase 1/2; NCT04570644
atabecestat	BACE1I	367	N = 5	Discontinued
avagacestat	GSI	520	N = 5	Discontinued
BI1181181	BACE1I	n/a	n/a	Discontinued
ELND005	Blocks Abeta oligomers	180	N = 12	Inactive
EVP0015962	GSI	450	N = 2	Trial completed
elenbecestat	BACE1I	437	N = 5	Discontinued
LY2886721	BACE1I	390	N = 5	Discontinued
LY3202626	BACE1I	499	N = 7	Discontinued
lanabecestat	BACE1I	412	N = 3	Discontinued
NIC5-15	GSM	194	N = 12	Trial completed
PF-06648671	GSI	539	N = 3	Discontinued
PF-06751979	BACE1I	456	N = 6	Discontinued
RG7129	BACE1I	389	N = 6	Discontinued,
semagacestat	GSI	361	N = 10	Discontinued
umibecestat	BACE1I	514	N = 6	Completed; NCT03131453
verubecestat	BACE1I	409	N = 8	Discontinued

BACE1I: beta secretase-1 inhibitor; GSI: gamma secretase inhibitor; GSM: gamma secretase modulator; the National Clinical Trial (NCT) number is from the Alzforum database [81].

**Table 2:** Therapeutic antibodies targeting amyloid-beta in clinical trials for Alzheimer’s disease.

Antibody	Mechanism	Target	Clinical Trial
bapineuzumab	Humanized 3D6 antibody	Abeta N-terminus	Trial terminated, NCT00112073
AAB-003	Bapineuzumab without effector function	Abeta aggregates	Phase 1 trial completed, NCT01193608
aducanumab	Human IgG1	Abeta aggregates	Phase 3 trial completed, NCT04241068
BAN2401	Humanized mAb158 antibody	Abeta proto-fibrils	Trial in phase 3, NCT04468659
crenezumab	Humanized antibody	Abeta aggregates	Trial terminated, NCT03491150
donanemab	Humanized mE8 antibody	Abeta(3–42)	Trial in phase 2, NCT04437511
GSK933776	Humanized antibody with reduced effector function	Abeta N- terminus	Trial terminated, NCT00459550
gantenerumab	Human IgG1 antibody	Abeta fibrils	Trial in phase 3, NCT04339413
LY2599666	Pegylated Fab antibody		Trial terminated, NCT02614131
LY3372993	antibody	Abeta	Trial in phase 1, NCT04451408
MEDI1814	antibody	Abeta(1–42) monomer	Phase 1 trial completed, NCT02036645
ponezumab	Humanized antibody	Abeta(33–40)	Trial terminated, NCT01125631
RO7126209	BSA of gantenerumab and monovalent Fab to TfRMAb	Abeta fibrils	Phase 1 trial completed, NCT04023994
SAR228810	Humanized antibody	Soluble Abeta fibrils	Phase 1 trial completed, NCT01485302
solanezumab	Humanized antibody	Mid Abeta domain	Trial terminated, NCT02614131

NCT number is from the Alzforum database [81].

**Table 3:** Therapeutic antibodies targeting Tau or TREM2 in clinical trials for Alzheimer’s disease.

Antibody	Mechanism	Target	Clinical Trial
ABBV-8E12	Humanized IgG4 antibody	Aggregated EC Tau	Trial terminated, NCT03712787
BIIB076	Human IgG1	Tau mid-domain	Phase 1 trial completed, NCT03067729
bepranemab	Humanized IgG4	Tau (235–246)	Phase 1 trial completed, NCT03464227
Gosuranemab	Humanized IgG4	NT of EC Tau	Phase 2 trial, NCT03352557
JNJ-63733657	Humanized IgG1	Phospho Tau	Phase 1 trial completed, NCT03375697
Lu AF87908	Humanized IgG1	Phospho Tau	Phase 1 trial, NCT04149860
PNT001	Monoclonal antibody	Phospho Tau	Phase 1 trial, NCT04096287
Semorinemab	Human IgG4	Tau NT	Phase 2 trial, NCT03828747
zagotenemab	Humanized antibody	Tau aggregates	Phase 1 trial, NCT03518073
AL002	Humanized antibody	Activates TREM2	Phase 1 trial, NCT03635047

NCT number is from the Alzforum database [81].
